# Liquid biopsy‐based diagnostic evaluation of hypermethylated CpG sites for ovarian cancer diagnosis

**DOI:** 10.1002/1878-0261.70277

**Published:** 2026-06-16

**Authors:** Deepa Bisht, Sameer Gupta, Amrita Chaurasia, Manisha Sachan

**Affiliations:** ^1^ Department of Biotechnology Motilal Nehru National Institute of Technology Allahabad Uttar Pradesh India; ^2^ Department of Surgical Oncology King George Medical University Lucknow India; ^3^ Department of Gynaecology and Obstetrics Motilal Nehru Medical College Allahabad India

**Keywords:** ARMS‐PCR, biomarker, hypermethylation, MethyLight method, ovarian cancer

## Abstract

Ovarian cancer is a heterogeneous gynaecological malignancy characterised by high mortality and an absence of reliable biomarkers for detection. In this study, a CpG‐specific, ARMS‐PCR approach was employed to evaluate the methylation status of six diagnostically relevant CpG sites in 65 epithelial ovarian cancer tissues and 35 healthy controls. Based on methylation frequency, the top three CpG sites were selected and evaluated in two diagnostic panels. A TaqMan‐based MethyLight assay incorporating cg02957270, cg00480298 and Col2A1 (as endogenous control) was developed for tissue and serum cell‐free DNA cohort analysis. ARMS‐PCR demonstrated diagnostic sensitivities of 80%, 73.3% and 82.3% for singleplex and multiplex panels, respectively. However, the multiplex MethyLight assay achieved 86% sensitivity and 90% specificity, with an AUC of 0.97 in the serum cohort. Furthermore, while ARMS‐PCR panels displayed limited clinicopathological correlations, MethyLight showed significant correlations (*P* < 0.05). Overall, this pilot study highlights the promise of liquid biopsy‐based diagnostics using independent hypermethylated and hypomethylated CpG biomarkers for ovarian cancer detection.

## Graphical abstract

This schematic outlines the workflow from biomarker identification to duplex MethyLight assay validation for epithelial ovarian cancer diagnosis using cfDNA‐based liquid biopsy. Initial screening of hypermethylated CpG candidates (cg02957270, cg10061138 cg00480298, COL2A1) was performed in tissue using ARMS‐PCR, COBRA, qPCR and image analysis. Selected markers were further assessed through duplex MethyLight qPCR in tissue and serum cfDNA cohorts for validation.

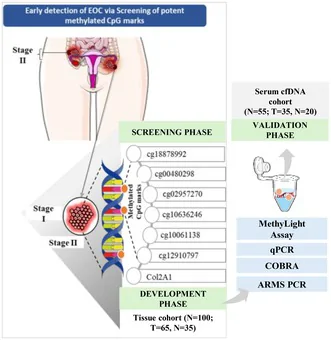

**Abbreviations**
ARMS‐PCR: Amplification Refractory Mutation System PCR
CA125: cancer antigen 125
cfDNA: cell‐free DNA
COBRA: Combined Bisulfite Restriction Analysis
ENCODE: Encyclopedia of DNA Elements
EOC: Epithelial ovarian cancer
HGS: High‐grade serous carcinoma
MSP: Methylation‐specific PCR

## 1 Introduction

Ovarian cancer ranks as the third most prevalent gynaecological malignancy in India and the eighth globally, yet it remains one of the most challenging diseases for detection. Ovarian cancer's asymptomatic progression and heterogeneity lead to over 70% of cases being diagnosed at later stages (III–IV), with a 5‐year survival rate of around 30% [1, 2]. Epithelial ovarian cancer (EOC) marks ~ 90% of ovarian malignancies and comprises serous, mucinous, endometrioid and clear cell subtypes. High‐grade serous carcinoma (HGS), the most aggressive and lethal subtype, accounts for ~ 75% of EOC cases [3]. Clinicians mainly detect ovarian cancer using transvaginal ultrasound, pelvic examination and serum biomarkers like cancer antigen 125 (CA125) [4], human epididymis protein 4 (HE4) [5], often linked with diagnostic algorithms risk of ovarian malignancy algorithm (ROMA) [6], risk of malignancy index (RMI) [7], ovarian malignancy algorithm 1 (OVA1) [8], Overa (OVA2) and International Ovarian Tumour Analysis Models (IOTA) [9]. Routinely symptomatic cases undergo surgical biopsy for definitive diagnosis and therapy; however, routine population screening is not done due to the limited sensitivity and specificity of present methods [10, 11].

The human genome harbours ~ 28 million CpG sites, with around 80% of these cytosines typically methylated [11]. In cancer, an abnormal DNA methylation profile is established by region‐specific hypermethylation at CpG‐rich areas regulating tumorigenesis and its progression; notably, tumour cells of the same type often exhibit consistent, distinct methylation profiles [12]. Early and stable hypermethylation marks in cell‐free DNA (cfDNA), detectable via liquid biopsies, offer superior stability, real‐time monitoring and high cellular specificity compared to RNA and proteins, making them robust, minimally invasive biomarkers for cancer diagnosis [13, 14]. DNA methylation biomarkers initially focused on hypermethylation of CpG islands in tumour suppressor gene promoters, but Koch et al. (2018) pointed out that the functional and diagnostic relevance varies across independent CpGs, highlighting the need to review biomarkers' genomic locations for higher accuracy [15]. With these basics, we have shortlisted six independent CpG sites that are strongly associated with a high risk of epithelial ovarian cancer occurrence. We propose that multiplexing multiple CpG sites simultaneously enhances the clinical biomarker potential by improving detection sensitivity and specificity.

Researchers have studied various tissues, blood, vaginal fluid, serum and other methylation‐based multi‐biomarker panels, suggesting improved sensitivity and specificity for early detection [1, 16]. There are various methods for detecting DNA methylation at specific loci, including Methylation‐Specific PCR (MSP) [17], Digital Droplet PCR (ddPCR) [18], MethyLight and digital MethyLight [19], MethyQuant [20], Epityper [21], Methylation‐Sensitive Restriction Enzyme‐Based Approaches (MSRE) [22], MSRE‐PCR [23], Combined Bisulfite Restriction Analysis (COBRA) [24], Methylation‐Sensitive High‐Resolution Melting (MS‐HRM) [25], and new next generation sequencing (NGS) based approaches [26]. These techniques enable precise analysis of DNA methylation patterns, but their resolution, specificity, sensitivity, quantitative nature and accuracy vary [6, 27, 28]. MSP and MethyLight are sensitive, specific and quantitative PCR‐based assays suitable for precise detection of methylation‐specific biomarkers in cancer diagnostics.

**Fig. 1:**
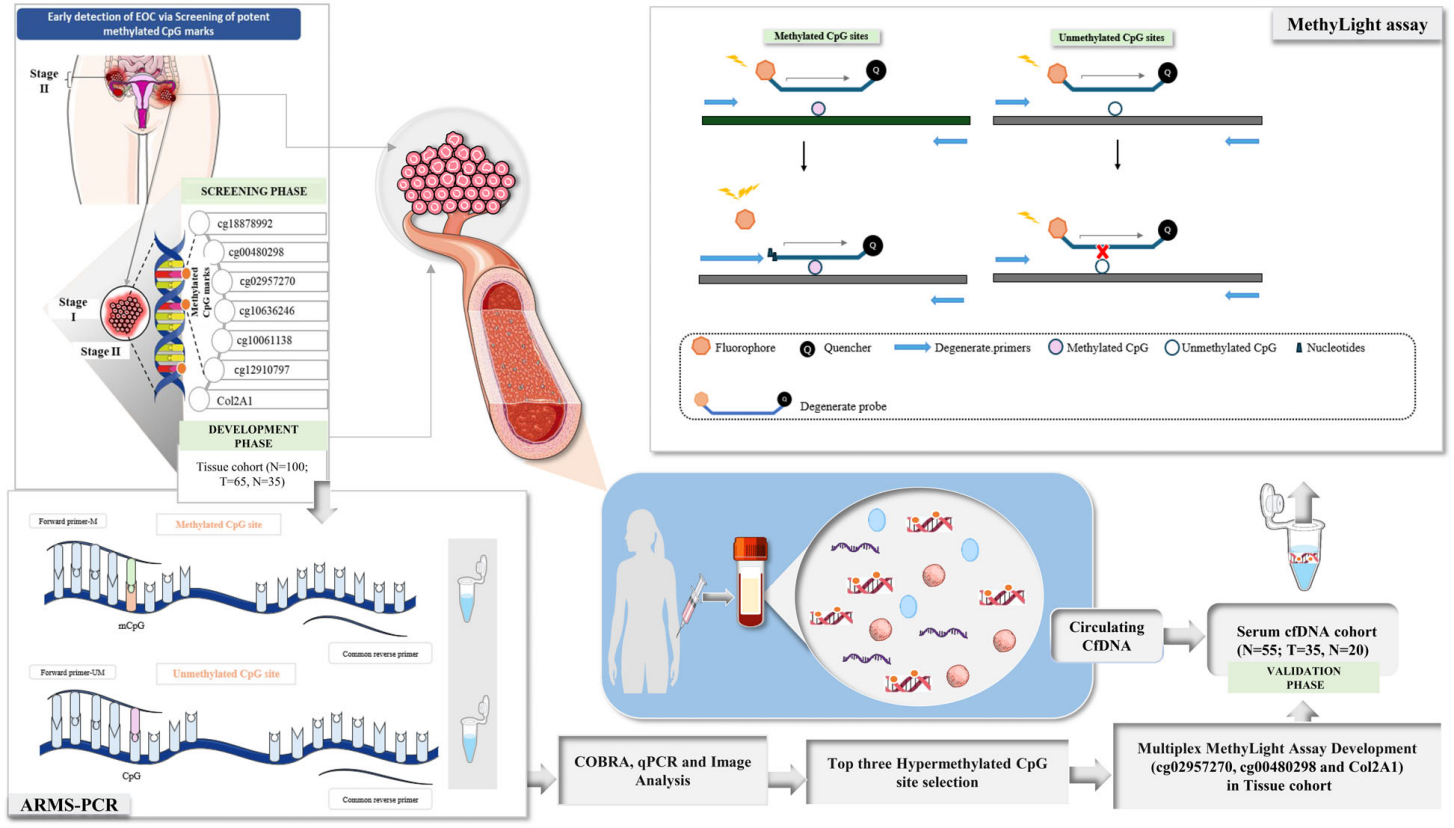
Workflow from biomarker identification to duplex MethyLight validation for epithelial ovarian cancer using cfDNA‐based liquid biopsy. Hypermethylated CpG candidates (cg02957270, cg10061138, cg00480298, *COL2A1*) were screened in tissue using ARMS‐PCR, COBRA, qPCR, and image analysis, then validated in tissue (*N* = 100) and serum cfDNA (*N* = 55) via duplex MethyLight qPCR. This Figure was partly adapted from Serveir Medical art (https://smart.servier.com), licensed under CC BY 4.0 (https://creativecommons.org/licenses/by/4.0/).

Building on Tian et al. [29], who showed the importance of Amplification Refractory Mutation System PCR (ARMS‐PCR) for CpG methylation detection, we tailored this technique for ovarian cancer methylation analysis to develop a variant of MSP. By employing dual forward primers with 3′ ends specific to methylated and unmethylated cytosines and one common reverse primer, this method enables precise, sensitive and easy single‐CpG methylation profiling for preliminary biomarker evaluation [29, 30]. To address limitations of ARMS‐PCR, we applied a high‐throughput quantitative MethyLight assay to assess methylation at the top three hypermethylated CpG sites. This real‐time fluorescence‐based method uses methylation‐specific TaqMan probes for sensitive and reproducible locus‐specific quantification [31]. The primary aim of the present study is to develop a rapid, sensitive and specific assay for the concurrent detection of multiple individual CpG sites to assist in the diagnosis of EOC. The selected CpG targets and the schematic representation of the overall workflow are presented in Fig. 1.

## 2 Material and methods

### 2.1 Patients and clinical sample collection

Ovarian cancer tissue samples were obtained from a cohort of consenting patients aged 22–65 years, who were enrolled for treatment at the Surgical Oncology Department of King George's Medical University, Lucknow, with the understanding and written consent of each subject. Simultaneously, normal tissue samples were gathered from the ovaries of patients who underwent cystectomy at the Swaroop Rani and Motilal Nehru Medical College, Prayagraj. The study encompassed 100 tissue samples (65 from EOC cases and 35 from healthy subjects) and 55 matched serum samples (35 from EOC patients and 20 from normal controls). The study received ethical (IEC No. IEC/2021‐22/05) and biosafety (IBSC Approval No. MLNNIT‐1100) approvals from MNNIT Allahabad institutional committees. Clinicopathological data for the patients, including age, tumour size, CA125 levels, menopausal grade, cancer histotype and FIGO staging, were precisely collected from both medical and pathological records. Promptly after surgery, all tissue samples were collected and rapidly stored at −80 °C for preservation and future analysis.

### 2.2 Selection of targeted CpG sites

Six CpG sites (cg18878992, cg029572270, cg00480298, cg10636246, cg10061138, cg12910797) with one endogenous control COL2A1 (collagen type II alpha 1 chain) lacking a CpG site were selected as potent methylation epi‐marks (Table 1). The primary selection criteria for CpGs were based on hypermethylation patterns observed at these specific loci and their strong association with EOC occurrence. Additionally, their inclusion in the study was validated by a comprehensive literature review, which consistently reported the significance of CpG sites (cg10636246 and cg10061138) [18, 32, 33, 34, 35]. The rest of the CpG targets from Yang et al., an extensive investigation involving a large cohort (63 000 European women), with consistent performance of the following four CpGs, viz., cg18878992, cg00480298, cg029572270 and cg12910797. Their integrative analytical research included examining methylation patterns, genetic markers and gene expression data. Their findings support the budding idea that these CpG sites, which are strongly associated with high EOC risk, can be used as targets in diagnostic assays [36]. It was further investigated that these CpGs have not been experimentally examined, and their methylation scores in OVCAR3 cell lines were assessed from the Encyclopedia of DNA Elements (ENCODE) database (RRID:SCR_006793), as shown in Table 1. The regulatory network for these CpGs has already been reported for breast cancer via the CanMETH database (SCR_023947). In the MethyLight assay, we selected the three most hypermethylated CpGs identified by ARMS‐PCR (cg02957270, cg00480298 and cg10061138) for multiplex assay development. Subsequently, we analysed cg02957270 and cg00480298 in the serum cfDNA cohort.

**Table 1:** Overview of CpG sites with their methylation score and designed primers for ARMS‐PCR test with weak mismatch for multiplexing; Y=C, T, R = G, A Yellow highlight‐mismatches, Grey highlight‐degeneracy.

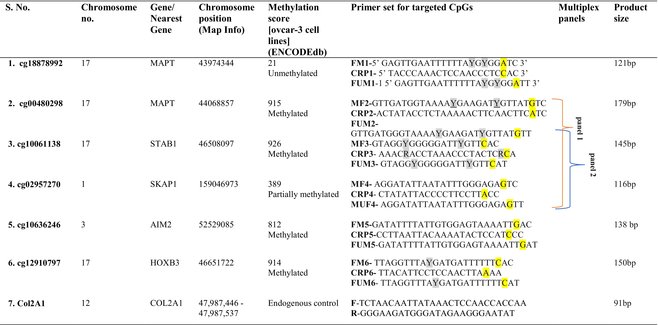

### 2.3 DNA isolation and bisulfite conversion

DNA isolation was performed from 10 mg of fresh frozen tissue using the SDS‐proteinase K method. The bisulfite‐treated DNA was obtained by modifying 500 ng of genomic DNA using a Premium Bisulfite kit (Diagenode, Seraing, Belgium; Cat. No. C02030030). To assess the effectiveness of bisulfite‐treated DNA (Bs DNA), PCR was conducted using COL2A1 gene. Cell‐free DNA (cfDNA) was obtained from 700 μL of serum using the EpiQuik Circulating cfDNA Isolation Kit (EpiGentek, Farmingdale, NY, USA) as per the user guide. Extracted cfDNA was eluted in 20 μL elution buffer, quantified by Qubit® 2.0 Fluorometer and detailed cfDNA fragment analysis using Agilent 2100 Bioanalyzer, yielding around 45 ng DNA per 700 μL of serum. This cfDNA is then converted to bisulphite cell‐free DNA (Bs CfDNA) using Premium Bisulphite kit (Diagenode Cat. No. C02030030).

### 2.4 Methylation‐specific ARMS‐PCR method

#### 2.4.1 CpG‐specific primer designing

Degenerate primers were created and assessed employing the ARMS method. Designing was performed using MethPrimerDB (RRID:SCR_012017), and validation was performed using Primer Premier 6.25 (RRID:SCR_023946) and Primer‐BLAST (RRID:SCR_003095). The methylation status of targeted CpGs was measured using a novel CpG‐specific ARMS‐PCR, which combined a weak mismatch at the antipenultimate primer position and degeneracy for nontargeted CpGs to enhance amplification specificity. Therefore, for each CpG, a set of three primers: the forward methylated (F‐M) primer targets the methylated CpG state, the forward unmethylated (F‐UM) primer targets the unmethylated CpG state, and the common reverse primer (CRP) binds downstream of the bisulfite‐changed sequence. The primer sequences for all targeted CpG sites are listed in Table 1.

#### 2.4.2 Standardisation of reaction components and annealing temperatures for singleplex and multiplex ARMS‐PCR method

All targeted CpG sites were amplified with endogenous control separately for methylated and unmethylated alleles in a standardised reaction volume of 20 μL containing 2 μL of (10X PCR buffer; QIAGEN), 1.0 μL of BsDNA (33 ng·μL^−1^), 1.5 μL of the forward [F‐M F^−1^‐UM (10 pmol·μL^−1^)] and reverse primers (CRP) (10 pmol·μL^−1^), 1 μL each forward and reverse primer of COL2A1 (10 pmol·μL^−1^), 3 μL of dNTPs (2.5 mM each), 0.1 μL HotStarTaq DNA Polymerase (5 U·μL^−1^, QIAGEN), 0.4 μL of 25 mm MgCl_2_ (QIAGEN) and 8.5 μL of Nuclease‐free water. The gradient PCR protocol was performed to optimise the annealing temperature for all targets across the 50 °C–62 °C range using a BIORAD S1000™ thermal cycler. Among the top three hypermethylated CpG targets, two duplex panels (Panel 1: cg02957270 + cg00480298 + COL2A1; Panel 2: cg02957270 + cg10061138 + COL2A1) were created with the reaction volume of 25 μL with increased concentration of the BsDNA, HotStarTaq DNA Polymerase and 2.5 mm MgCl_2_. The following reaction program was used: 95 °C for 15 min, followed by 40 cycles of denaturation at 95 °C for 30 s, annealing at 50 °C–62 °C for 30 s and final extension at 72 °C for 30 s. A final extension was finished at 72 °C for 10 min, and the reaction was then kept at 4 °C for 5 min. All bisulfite‐converted tumour and normal cohort samples were checked for methylation status at an optimised annealing temperature for the targeted singleplex and multiplex CpG panels.

#### 2.4.3 Agarose gel electrophoresis and image analysis using IbrightImager

The amplified PCR products were resolved on 3% agarose gels and visualised using a gel documentation imaging system (iGene Labserve). To assess differential band intensity between methylated and unmethylated amplicons, gel images were further analysed for three CpG sites cg02957270, and cg10061138 representing hypermethylated loci, and cg18878992, representing a hypomethylated locus. Quantitative pixel‐intensity measurements were acquired with the iBright™ Imaging Systems (Thermo Fisher Scientific, Waltham, MA, USA) to confirm a discriminative pixel intensity between methylated and unmethylated bands.

#### 2.4.4 COBRA and qPCR analysis

The COBRA (Combined Bisulfite Restriction Analysis) method was used to validate methylation at two CpGs, cg00480298 and cg02957270, which have overlapping BstU1 sites. This enzyme specifically cleaves at the CG/CG restriction site with methylated cytosines on the targeted CpGs. Following bisulfite treatment, restriction digestion is carried out using the BstUI enzyme at 60 °C for 1 h with 10 μL PCR product, 2 μL (rCutSmart™ Buffer 10X), 0.5 μL BstU1 enzyme (10 000 units/mL), and 7.5 μL RNase‐free water. The top three potent discriminatory CpG sites (cg00480298, cg02957270; hypermethylated), (cg18878992; hypomethylated) allele quantification was performed by quantitative PCR (qPCR) in a standardised reaction volume of 10 μL comprising 5 μL of PowerUp™ SYBR™ Green Master Mix (Thermo Fischer Scientific), 1.0 μL of Bs DNA (33 ng·μL^−1^), 0.5 μL each of both primers (10 pmol·μL^−1^), and 3.0 μL of Nuclease‐free water. The methodology was systematically applied to the entire set of 100 tissue samples at an optimised annealing temperature using the QuantStudio 3 instrument (Applied Biosystems, Waltham, MA, USA). Then, the subsequent reaction program was followed: 95 °C for 15 min; 95 °C for 15 s, optimised annealing temperature, 30 s for 40 cycles, and standard melt curve program.

### 2.5 MethyLight method

#### 2.5.1 Primer and probe designing

For the TaqMan‐based method, degenerate primers and fluorescently labelled probes were specifically designed by IDT (Integrated DNA Technologies, Coralville, IA, USA) for cg00480298, cg02957270 and cg1006113, as shown in Table S1. The amplicon length was minimised to optimise efficiency and specificity in the multiplex qPCR assay. COL2A1 (endogenous reference gene) was standardised for input DNA. The primers and probe targeting COL2A1 were precisely designed from a CpG‐free promoter region to ensure consistent amplification regardless of methylation status.

#### 2.5.2 Standardisation of reaction parameters for singleplex and multiplex methods

The MethyLight method was conducted in duplicates using an AriaMx Real‐time PCR Instrument in a 20 μL total reaction volume encompassing 2 μL of BsDNA (33 ng·μL^−1^), 0.5 μL both forward and reverse primers (10 pmol·μL^−1^) for each CpG site and Col2A1, 0.2 μL probe (10 pmol·μL^−1^), and 10 μL 2 × Prime time Gene expression master mix (IDT) and remaining nuclease‐free water. The protocol followed was: 95 °C for 4 min, then 50 cycles of denaturation at 95 °C for 15 s, with annealing or extension at optimised temperature for 35 s. The multiplex MethyLight method was performed using the same sequences and optimised PCR settings for both the tissue (cg00480298, cg02957270 and cg1006113) and serum (cg00480298, cg02957270) cohorts. The mean Ct value from duplicate experiment was further employed for data analysis. A fully methylated positive control was made by *in vitro* treatment of normal ovarian genomic DNA with M.SssI methyltransferase (NEB), followed by bisulfite conversion and storage at −80 °C for subsequent analysis.

#### 2.5.3 PMR calculation

The Percent of Methylated Reference (PMR) value at a precise point was determined by calculating the ratio of methylation levels of the target gene, standardised to the control gene, compared to the methylation levels observed in the M.SssI‐treated sample.

Based on literature, a cutoff value of 4 was used to classify the methylation levels of the CpG loci. PMR values above and below thresholds were classified as methylated and unmethylated, respectively. PMR values were calculated for both singleplex and multiplex data using the standard formula [37, 38, 39].

### 2.6 Statistical analysis

Statistical analysis was performed to study patterns of DNA methylation and various clinical features of the patient. This analysis involved the application of the Mann–Whitney U test, chi‐square, and Student's *t*‐test sensitivity and specificity calculation using standard formulas (ARMS‐PCR), while utilising a mathematical model for MethyLight. Relative fold change was determined using the Livak method and PMR calculation using Microsoft Excel 365. Graphs were formed using GraphPad Prism V 9.0 and Microsoft Excel 365. A receiver operating characteristic (ROC) curve investigation was conducted to assess the discriminatory power of hypermethylated CpG in distinguishing EOC vs control, using individual PMR values for singleplex and combined PMR values for multiplex. Data visualisation using heat maps, ROC curves, and the area under the curve (AUC) was performed using the Python programming language (version 3.9.12). The optimal cutoff value, which maximised the sum of (sensitivity + specificity − 1), was determined using logistic regression (singleplex) and multiple logistic regression (multiplex) methods. All statistical evaluations were two‐sided, with significance *P* < 0.05.

## 3 Results

### 3.1 Methylation analysis of six CpG sites with ARMS‐PCR method

#### 3.1.1 Methylation status of singleplex and multiplex CpG targets employing ARMS‐PCR

Fig. 1 summarises the workflow from biomarker identification to duplex MethyLight assay development for EOC using serum cfDNA. ARMS‐PCR (cg18878992, cg00480298, cg029572270, cg10636246, cg10061138, cg12910797) and COBRA are initially employed on the tissue cohort (*N* = 100 T‐65, N‐35), leading to the selection of cg02957270, cg00480298 and cg1006113 as the top hypermethylated CpG sites. Subsequently, serum cfDNA was isolated and quantified; following tissue cohort testing, serum cfDNA validation was performed (*N* = 55 T‐30, *N* = 25) using a TaqMan‐based MethyLight assay. The preliminary methylation score for these sites was derived from the ENCODE database, as summarised in Table 1.

Figure S1A–H depicts PCR amplification results at different optimised annealing temperatures, 52 °C, 54.5 °C, 52.4 °C, 54.5 °C, 54.3 °C and 55 °C, respectively, corresponding to amplicon size for each target. The three most significantly hypermethylated genomic loci are depicted as cg02957270, cg00480298 and cg10061138. Conversely, the site displaying notable hypomethylation is cg18878992. Among the top three significantly hypermethylated CpG sites, two multiplex panels were devised, as outlined in Table 1 and depicted in Fig. S1I,J. The optimised annealing temperatures for the multiplex panels were 54 °C for panel 1 and 53.7 °C for panel 2. Panel 2 (cg02957270 and cg10061138) showed superior discriminatory capacity, providing more distinct and selective insights into methylation status by efficiently differentiating between methylated and unmethylated alleles in combination.

However, the application of this rapid CpG‐specific PCR method yielded mixed results, with approximately 50% of both tumour and normal samples showing partial methylation patterns. This percentage‐wise data were reflected in Table S2, where individual CpG site amplicon generation took place in both methylated and unmethylated alleles.

#### 3.1.2 Post‐PCR partial methylation profile analysis

**Fig. 2:**
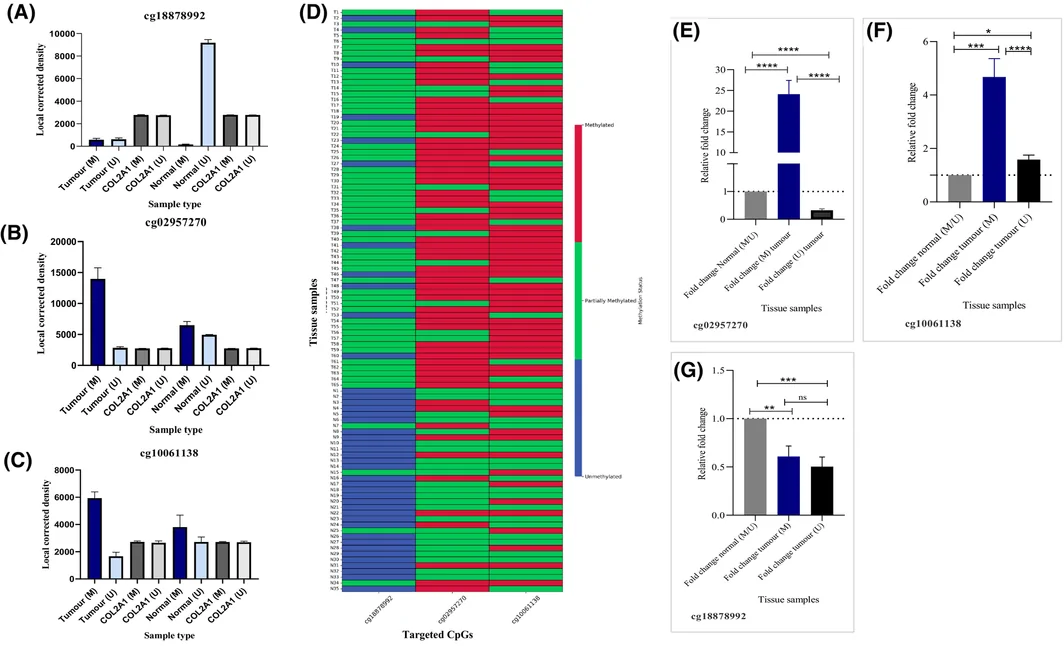
Image Analysis and qPCR based allele quantification of top hypermethylated CpGs. Image analysis Mean ± SD of (A) cg18878992, (B) cg02957270 and (C) cg10061138 targets showing differential pixel intensity of methylated (M) and unmethylated (U) alleles in gel bands of (T‐3, N‐3) and Mann–Whitney test showing *p* = ns in M vs U tumour and normal samples. (D) Heatmap showing differential methylation status (methylated, unmethylated and partially methylated) of top two hypermethylated (cg02957270, cg10061138) and hypomethylated (cg18878992) targets in different tissue samples *N* = 100 (*T* = 65, *N* = 35); Column bar graph representation showing distribution of methylated/unmethylated allele using qPCR analysis for CpG sites (E) cg02957270 and (F) cg10061138, and (G) cg18878992 in *N* = 100 (*T* = 65, *N* = 35) with unpaired *t*‐test between normal and tumour M/U alleles.

To assess the partial methylation patterns, we performed both Image analysis and COBRA to confirm the results of our PCR amplifications. Image analysis of explicitly hypermethylated target sites: cg02957270 and cg10061138, as well as hypomethylated cg18878992, exhibited noticeable variations in local corrected density post background pixel correction in a similar trend as shown in Fig. 2A–C. Image analysis and COBRA validated the PCR results, including partial methylation patterns, showing distinct variations in local corrected density for hypomethylated (cg18878992) sites Fig. 1A and hypermethylated (cg02957270, cg10061138) Fig. 2B,C and consistent with the observed methylation trends. The methylation state was defined as 100% for fully methylated alleles, 0% for unmethylated alleles and 1%–99% for partially methylated alleles across the designated targets. The distribution of these methylation levels within the tissue cohort was systematically visualised through a heatmap, illustrating the gradation of methylation patterns across samples Fig. 2D.

A corresponding proof of this information can be seen in the COBRA results in Fig. S1G,H which show two significant CpGs with BstU1 restriction sites. The digestion of cg00480298 (150, 28 bp) yielded a distinct cleavage pattern of methylated and unmethylated alleles. It validates true methylation of PCR products. On the contrary, the digestion results for cg02957270 explained partial methylation patterns (116 bp, 88 bp, 28 bp), interpreted as the concurrent presence of both intact PCR amplicons and digested fragments. This partial methylation pattern marks the coexistence of both alleles (M/U) and specifies the heterogeneity of the tissue sample.

#### 3.1.3 Quantitative PCR and clinicopathological correlation of ARMS‐PCR

Quantitative analysis via qPCR confirmed the methylation status of precise CpG sites, namely cg02957270 and cg10061138, as hypermethylated, while cg18878992 as hypomethylated Fig. 2E–G. Statistical analysis employing unpaired Student *t*‐methods revealed noteworthy findings. In the case of cg02957270, a highly significant correlation was observed between methylation levels in the tumour and normal samples (*P* < 0.0001). Additionally, a noteworthy correlation was identified between the relative quantitative levels of methylated and unmethylated alleles in tumour samples (*P* < 0.0001). Similarly, for cg10061138 and cg18878992, a significant association between methylation levels in tumour and normal samples was observed, with *P*‐values <0.0001 for both sites. However, in contrast, for cg18878992, the quantitative levels of the unmethylated allele exhibited very high significance (*P* < 0.0001), while there was no noteworthy correlation detected between the quantifiable levels of the methylated and unmethylated alleles in tumour samples of cg18878992. The singleplex panels targeting hypermethylated loci showed sensitivities of 80% and 73.3%, respectively, with significant association only to tissue type (*P* < 0.05). In contrast, the multiplex panel (cg02957270 + cg10061138) exhibited enhanced sensitivity (82.3%) and a strong correlation with tumour versus normal samples, independent of other clinicopathological features (Table S3).

### 3.2 Methylation analysis of the top three CpG sites with MethyLight method

The methylation levels of the top three hypermethylated CpG sites (cg02957270, cg00480298 and cg1006113, verified by ARMS‐PCR and COBRA) were accurately quantified using MethyLight, a sensitive fluorescence‐based real‐time PCR assay for locus‐precise methylation detection. The optimised annealing temperature in the singleplex format was 60 °C for cg02957270, cg00480298, including COL2A1, but 55 °C for cg1006113. So, duplex targets (cg02957270, cg00480298 with COL2A1) were checked for methylation frequency across different tissue cohort samples and serum cfDNA at an optimised annealing temperature of 60°. The one‐dimensionality and accuracy of the technique were estimated by performing a sequential dilution of a positive reference standard with a known concentration of input DNA. Standard curves for COL2A1, cg02957270 and cg00480298 were created using MSssI‐treated normal tissue samples in a multiplex PCR format Fig. S2A–C. The standard curves generated *R* ^2^ values of 0.9791, 0.9667 and 0.9904, respectively, suggesting good replicability across standards from diverse samples. Amplification plots were generated for the multiplex panel in both serum and tissue cohorts Fig. S3 using the TaqMan‐based technique.

**Fig. 3:**
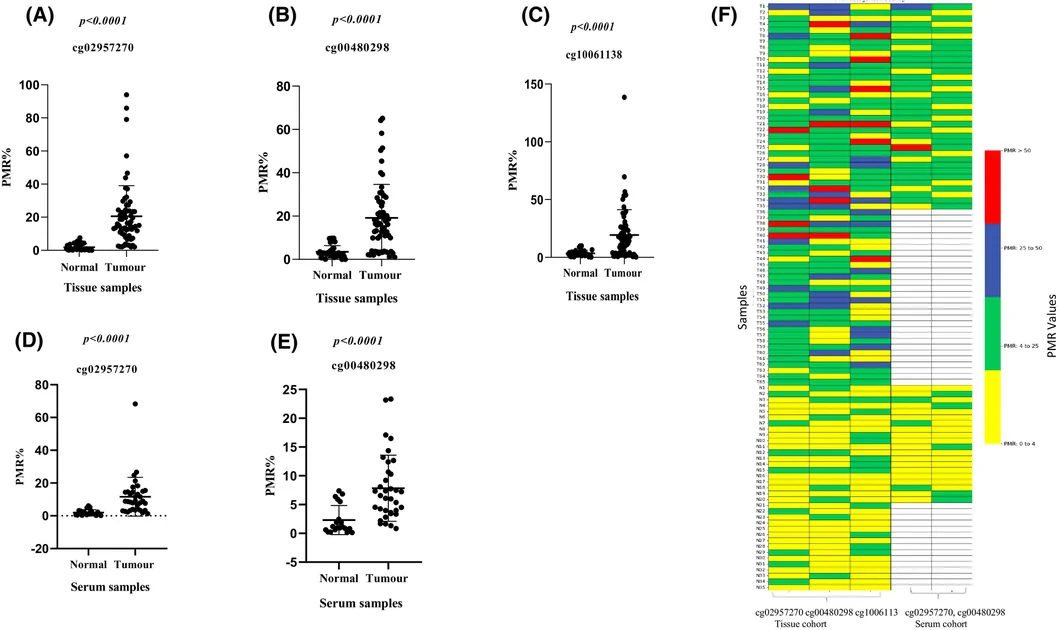
Distribution of PMR values with SD in EOC and normal tissue samples. Single plex panels (A) cg02957270, (B) cg00480298 and (C) cg1006113; Serum samples (D) cg02957270 (E) cg00480298 whereas representative (F) Heat map illustrating the distribution of PMR values for the same CpGs. The heat map illustrates the methylation levels in tissue DNA from 65 EOC samples and 35 control samples along with serum samples *N* = 55 (*T* = 35, *N* = 20). The colour scale represents the range of Percent Methylation Ratio (PMR) levels. Abbreviation: PMR: percentage of methylated reference.

Correlation coefficients (*R* ^2^) of (0.78, 0.57) and (0.61, 0.81) for cg02957270 and cg00480298, respectively, for the tissue and serum cohorts specify that the newly developed multiplex MethyLight assay performs comparably to singleplex methods in assessing methylation in EOC samples. Methylation frequencies were assessed for cg02957270, cg00480298 and cg1006113 in 65 EOC tissue samples, mainly of the serous subtype, compared with 35 normal ovarian samples. The PMR value, which enumerates the ratio of fully methylated fragments at a precise locus, was used for evaluation. The dispersal of PMR values is depicted in Fig. 3A–C in tissue and Fig. 3D,E in serum and pictorially through a heatmap in Fig. 3F.

The mean and median PMR values for all three CpGs (cg02957270, cg00480298 and cg10061138) are (21.22 ± 2.39, 15.6; 20.87 ± 2.23, 15.95; 19.26 ± 2.86, 13.9) for EOC vs (2.36 ± 0.32, 1.35; 3.71 ± 0.47, 2.88; 3.47 ± 0.53, 2.44) for healthy control, respectively. The majority of EOC samples showed PMR values greater than 4 for both the CpGs in the range of (2.03–93.95), (0.95–65.06) and (0.25–138.51), respectively (Table S4). The PMR values indicate methylation of the CpGs is significantly higher than in matched normal controls, with *P*‐values < 0.0001 for all three CpGs and also for the multiplex method. Also, the mean and median PMR values for two CpGs (cg02957270 and cg00480298) in serum cfDNA were 11.97 ± 1.95, 8.84 and 7.69 ± 0.86, 7.38, respectively, for EOC, compared to 2.04 ± 0.37, 1.64; 2.35 ± 0.49, 1.55 control samples. Most EOC serum samples also showed PMR values greater than 4, within the ranges of 1.25–68.30 for cg02957270, 0.83–23.33 for cg00480298, and corresponding methylation levels significantly elevated compared to normal controls (Table S4).

**Fig. 4:**
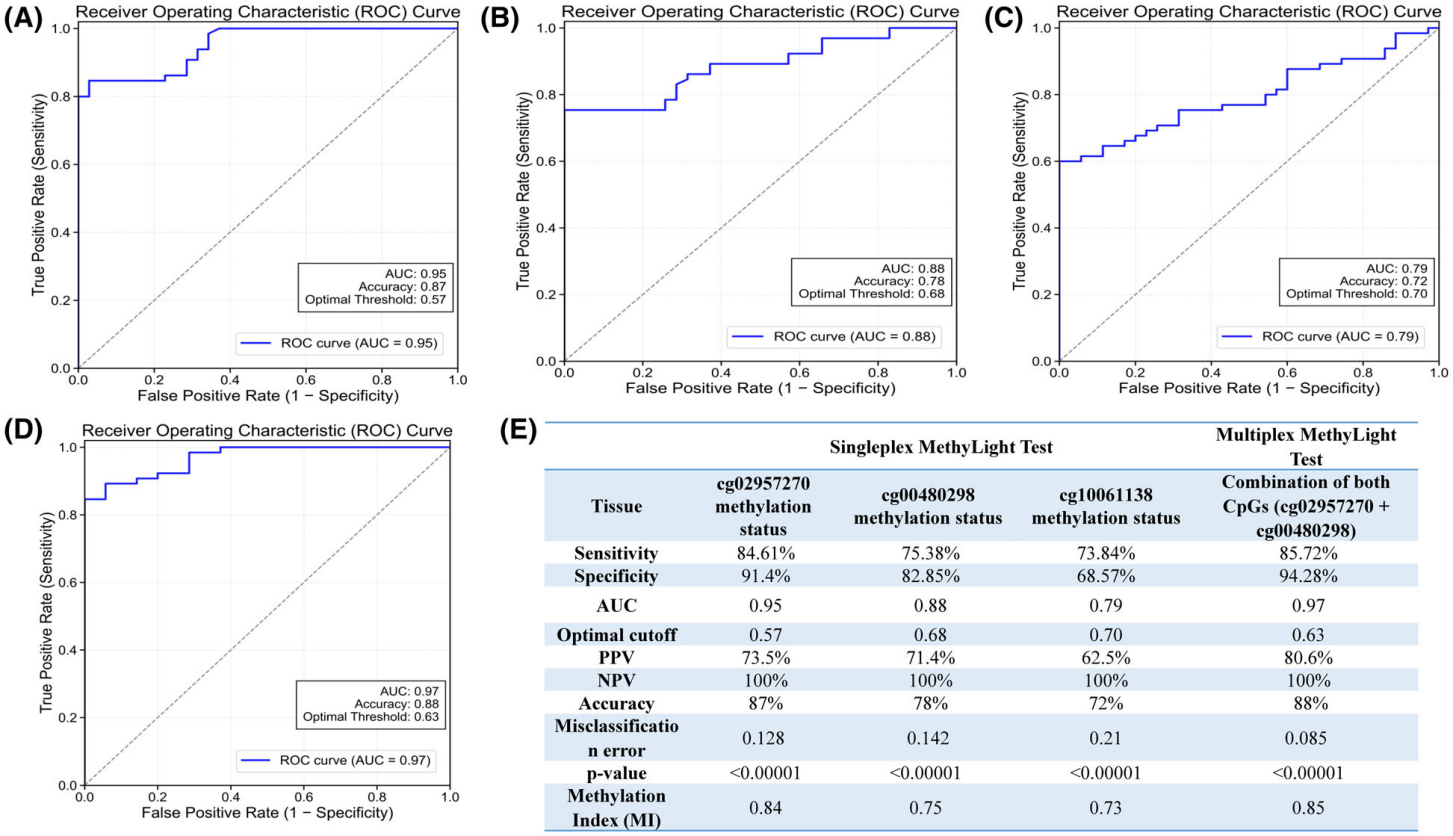
The receiver operating characteristic (ROC) curve analysis was conducted to differentiate EOC patients from healthy samples. ROC curves (A) cg02957270, (B) cg00480298, (C) cg10061138 and (D) combination of both CpGs (cg02957270 + cg00480298) (based on the sum of the two PMR values) in recognising 65 ovarian carcinoma from 35 healthy control samples (*N* = 100). (E) illustrates the diagnostic significance of the CpG based DNA methylation markers. This table presents specificity, sensitivity, optimal cutoff values, AUC area under the curve, PPV‐positive predictive value; NPV‐negative predictive value, accuracy and methylation indices for both singleplex vs multiplex MethyLight tests.

**Fig. 5:**
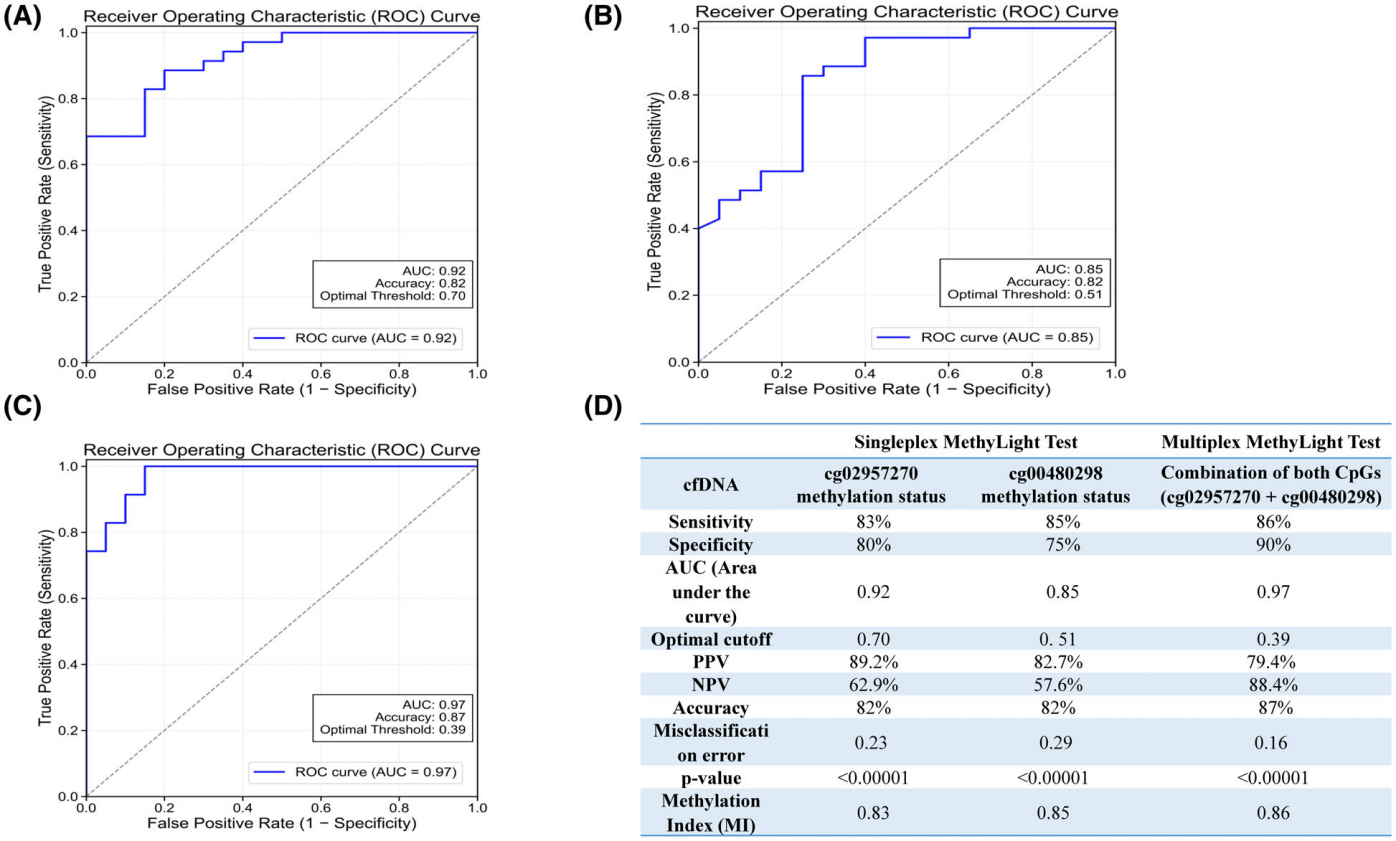
ROC curve analysis was directed to differentiate EOC patients from healthy samples using serum cell‐free DNA samples. The area in the ROC curve (AUC) values indicate the accurateness of the biomarkers (A) cg02957270, (B) cg00480298, (C) Combination of both CpGs (cg02957270 + cg00480298) (based on the combination of the two PMR values) in distinguishing 35 ovarian from 20 healthy control samples (*N* = 55). (D) Illustrates the diagnostic significance of the CpG‐based DNA methylation markers. This table presents specificity, sensitivity, AUC, optimal cutoff values, NPV, PPV, accuracy and methylation indices for both singleplex and multiplex MethyLight tests.

Utilising the MethyLight method, the sensitivity and specificity of singleplex cg02957270, cg00480298, and cg1006113 is 84.61%, 91.4%; 75.38%, 82.85% and 73.84%, 68.57%, respectively. When either or both CpGs (cg02957270, cg00480298) were methylated in multiplex format, the sensitivity and specificity were 85.72% and 94.28%, respectively. The ROC curve built using Machine learning logistic regression revealed AUCs of 0.95, 0.78 and 0.79 for the singleplex panels (cg02957270, cg00480298 and cg1006113) Fig. 4A–C. Moreover, an AUC of 0.97 was reported for the multiplex panel (cg02957270, cg00480298) in discriminating cancerous and normal samples (Fig. 4D). The optimal cutoff, NPV, PPV, accuracy, misclassification error and other parameters are reported in Fig. 4E. For the serum cohort, the combined sensitivity and specificity of the (cg02957270 + cg00480298) multiplex panel are 86% and 90%, respectively, with other parameters in Fig. 5A–D. Fig. 4E and Fig. 5D summarises all the diagnostic performance metrics, including specificity, sensitivity, AUC, accuracy, optimal cutoff and methylation indices of singleplex and multiplex MethyLight tests.

Clinicopathological assessment of the selected CpG loci revealed a highly significant association with tissue type and menopausal status; however, no meaningful correlation was detected with CA125 levels or patient age (Table S5).

## 4 Discussion

Our present study proves that hypermethylated CpG site‐based multiplex MethyLight assay enables a robust, rapid and quantitative approach for the detection of EOC using both tissue and matched serum cfDNA. Compared to conventional MS‐PCR, the developed assay exhibited superior sensitivity, specificity and reproducibility, highlighting its diagnostic precision. The integration of two hypermethylated loci, cg02957270 and cg00480298, along with the control gene Col2A1, markedly improved the diagnostic performance of the assay, achieving sensitivity and specificity values of 85.7% and 94.28% in the tissue cohort and 86% and 90% in the serum cohort, respectively. Its equivalence analysis in the serum cohort validates concordant methylation patterns at these loci and higher diagnostic potential as a minimally invasive assay.

MethyLight is a quantitative, TaqMan‐built real‐time PCR technique that detects DNA methylation at specific CpG sites with high sensitivity using methylation‐specific primers and probes. Our findings are consistent with previously reported data, confirming the reproducibility of the method, though differing in the selection of specific single‐CpG sites analysed [31]. In contrast to our study which focuses on independent hypermethylated CpG sites, earlier MethyLight applications in colorectal, prostate and epithelial ovarian cancers target multiple CpGs simultaneously of a CpG island in the promoter region. He et al. (2010) used multiplex MethyLight to detect SEPT9, ALX4, and TMEFF2 methylation in CRC: 56%, 78% and 75% sensitivity in tissues (48%, 75%, 71% in blood). Combined sensitivities reached 84% (tissue) vs 81% (blood), with 87% and 90% specificities. [40]. In another study SFRP2 methylation levels were 94.1% higher in tissues as compared to paired adjacent normal (*P* < 0.001). The sensitivity and specificity of SFRP2 for detecting CRC in serum were 69.4% and 87.3%, respectively [41]. Olkhov‐Mitsel et al. developed a multiplex MethyLight assay detecting HOXD3, APC, and TGFB2 hypermethylation in prostate cancer tissue, cell lines and urine with 100% specificity for methylated vs. unmethylated alleles demonstrating highly precise and simple method [42]. In EOC tissues, the multiplex MethyLight assay targeting HIC1, HOXA9 and SOX1 showed optimal sensitivity and specificity, effectively distinguishing cancer from healthy sera with AUCs of 0.95 (HOXA9 and HIC1), 0.93 (HIC1and SOX1) and 0.85 (HOXA9 and SOX1) respectively [43]. In another study by Singh et al., 2020, methylation of HOXA9 and HIC1 in 82.3% and 80.0% of ovarian tissue cohort, respectively, in singleplex format; in multiplex format, the sensitivity and specificity were 88.2% and 88.6% correspondingly with serum cfDNA showing sensitivity of 88.9% and AUC = 0.95 [39]. Ouchi et al. developed a modified MethyLight assay that stratifies metastatic colorectal cancer patients by DNA methylation grade, serving as a simple and predictive biomarker for anti‐EGFR treatment outcomes [44].

Although DNA methylation biomarkers hold transformative promise for revolutionising cancer detection, only Cologuard (stool DNA, NDRG4 and BMP3) [45, 46] and Epi proColon (blood, SEPT9) [47] have received FDA approval to date. Other commercial assays, including ConfirmMDx [48], EpiproLung [49], AssureMDx [50], PredictMDx [15] and Colvera [51] target various methylation signatures for the diagnosis or risk assessment of prostate, lung, bladder, glioblastoma and colorectal cancers, respectively. The commercialisation of DNA methylation biomarkers faces challenges due to the complex correlation between methylation patterns and their clinically significant genomic locations. Prominently, methylation of individual CpG dinucleotides, relatively than wider regions, critically influences gene expression and enhances the diagnostic and prognostic accurateness of these biomarkers; for example, methylation at +223 CpG in the ZAP70 gene correlates with better chronic lymphocytic leukaemia patient survival [52]. The defined genomic locus, as well as the requisite number of CpG sites to be incorporated for the biomarker assay, necessitates rigorous evaluation [15]. Single hypermethylated CpG biomarkers propose superior specificity and sensitivity by precisely targeting tumour‐specific methylation variations, reducing interference from other differentially methylated CpGs, and assisting simpler assays that enhance early cancer detection and clinical applicability.

In addressing this issue, we focused on validating the top six hypermethylated CpG sites associated with a higher EOC risk along with their consistent finding across the scientific literature. Among these six CpG sites (cg18878992, cg029572270, cg10636246, cg00480298, cg12910797 and cg10061138), only one CpG namely cg18878992, is located within a CpG island (chr17:43971411‐43975 040). These CpG sites are independent and have been correlated with the genes MAPT, STAB1, SKAP1, AIM2 and HOXB3, all of which are associated with essential functional and regulatory roles of cancer progression Table 1 [18, 32, 33, 34, 35, 36, 53, 54, 55].

Microtubule‐associated protein tau (MAPT), a pleiotropic regulator, acts as an oncogene in many solid cancers [56, 57] like breast [58], pancreatic [59], prostate [60], hepatocellular [61] and gastric cancers [62] and has a tumour‐suppressive role in certain central nervous system malignancies [63]. High MAPT expression correlates with aggressive disease, poor prognosis and resistance to taxane‐ and docetaxel‐based chemotherapy, driven by microtubule stabilisation and PI3K/AKT/mTOR activation [64], besides upregulation of Wnt/β‐catenin signalling [65]. Likewise, STAB1 (stabilin‐1) is a scavenger receptor that promotes immune evasion and tumour progression in high‐grade serous ovarian cancer [66], triple‐negative breast cancer [67], low‐grade glioma [68] and acute myeloid leukaemia mainly by driving immunosuppressive macrophage polarisation [69]. Its promoter hypermethylation is also linked to poor survival in glioma [68]. Likewise, SKAP1 (Src kinase‐associated phosphoprotein‐1) is an immune‐cell adaptor that regulates T‐cell integrin activation and immune‐synapse signalling, modulating antitumour immunity in different cancer types [70, 71, 72]. AIM2 (absent in melanoma 2) is a dsDNA‐sensing inflammasome nucleator that limits or promotes tumour progression by driving pyroptosis, cytokine release and immune‐microenvironment remodelling through both inflammasome‐dependent and independent signalling [73]. AIM2 plays a dual‐faceted role in ovarian cancer: it contributes to antiangiogenic therapy resistance, where elevated AIM2 inflammasome expression is associated with shorter progression‐free survival and reduced benefit from bevacizumab [74, 75]. In ovarian cancer, HOXB3 (Homeobox B3) is upregulated in high‐grade serous carcinoma and drives cisplatin and paclitaxel resistance by enhancing antioxidant responses and suppressing ferroptosis via the HOXA4/HOXB3 [76] and STUB1–HOXB3–PARK7 axes [77]. Looking at the deregulated expression of these genes in cancer, it warrants a comprehensive investigation of DNA methylation‐mediated gene regulation and its downstream signalling pathways governing ovarian cancer progression.

Our preliminary ARMS‐PCR identified three top hypermethylated CpGs (cg02957270, cg00480298, cg1006113), but faced limitations: degenerate primer Optimisation difficulties, inconsistent bisulfite‐DNA reaction conditions, poor reproducibility with low‐quality samples, qualitative detection lacking precise quantification and tissue heterogeneity causing partial methylation. We therefore adopted the more quantitative, reliable MethyLight assay for biomarker validation. [17, 78, 79, 80, 81, 82, 83]. To date, there is limited literature documenting the use of multiplex ARMS‐PCR for the detection of mutations or polymorphisms in cancer, and none has cited its use for methylation detection [84, 85]. One recent study by Wang et. al, 2020., pointed six co‐expressed differentially methylation sites (Co‐DMPs) (cg00134539, cg25268718, cg00226923, cg25839227, cg25697314, cg26574610) as potential ovarian cancer biomarkers by LASSO regression, and a diagnostic classifier utilising support vector machine (SVM) which could offer potential for early ovarian cancer diagnosis and personalised treatments [86]. Utilising machine learning, Luo et al. (2020) identified four CpG methylation markers that constitute an effective risk score model, offering valuable predictions for its recurrence, DNA damage response, clinical stage and potential immunotherapeutic efficacy, enhancing prognosis and treatment decisions in NSCLC [87]. A key limitation in existing studies is the lack of clinical sample validation, and notably, no reports have documented the use of single hypermethylated CpG epi‐marks either by ARMS‐PCR or MethyLight for diagnostic or predictive evaluation of ovarian cancer.

Multiplex ARMS‐PCR is a rapid approach for detecting DNA methylation at specific CpG sites with sufficient sensitivity and specificity using minimal instrumentation. Our assay targeting hypermethylated CpGs cg02957270 and cg10061138 showed promising potential for epithelial ovarian cancer detection but requires further optimisation to address challenges related to partial methylation patterns, specificity, sensitivity and technical issues [82]. Evidently, the method demonstrates sensitivities of 80% and 73.3% in singleplex panels and 82.3% in a multiplex ARMS panel. CpG multiplexing aims to explore methylation patterns at either of the two CpG sites of cancer samples when compared with normal samples. To quantify allelic methylation status at targeted CpG sites, we performed qPCR using SYBR Green chemistry. The findings indicate a statistically significant 23‐fold difference in the relative fold change between the methylated allele in tumour vs normal samples at the CpG cg02957270 locus (*P* ≤ 0.0001). Lower quantification levels were observed for the unmethylated allele, in contrast to the normal samples (*P* ≤ 0.0001), further substantiating the hypermethylated state of the CpG locus. When examining the other top two sites, cg10061138 and cg18878992, the methylated allele exhibited significant hypermethylation (*P* ≤ 0.001) and hypomethylation (*P* ≤ 0.01), respectively. However, the status of the unmethylated allele at cg18878992 was less conclusive, possibly due to tissue heterogeneity (Fig. 2E–G).

Our pioneering study introduces the first MethyLight assay targeting independent hypermethylated CpGs of exceptional diagnostic value, featuring a novel duplex panel (cg02957270, cg00480298, Col2A1) in serum cell‐free DNA. The combined panel achieved an outstanding ROC‐AUC of 0.97, powerfully distinguishing cancerous from normal samples.

## 5 Conclusion

This study proposes a novel approach to re‐examine autonomous CpGs and their multiplexing potential using simplified PCR‐based techniques, aiming to identify promising candidates for diagnostic and prognostic biomarkers. Moreover, it highlights the inadequacy of MSP as a suitable method for biomarker development. The study aims to re‐evaluate the diagnostic potential of individual CpG sites, emphasising the assessment of independent CpG methylation marks using a methylation‐sensitive qPCR‐based approach (MethyLight assay) as a proof‐of‐concept. One limitation of earlier methylation‐based methods is the consistency of methylation frequency at a specific CpG site within a region across different clinical samples. Designing methylated/unmethylated probe sets for a single‐CpG site is easy and technically simpler due to consistent methylation, reduced sequence complexity and lesser heterogeneity challenges than targeting multiple CpGs in a region.

Additionally, the study highlights the critical importance of hypermethylated CpG sites, including cg02957270, cg10061138 and cg00480298, in the diagnosis of epithelial ovarian cancer. Our ongoing pilot study signifies an initial stride toward establishing the importance of screening and validating autonomous hypermethylated CpG markers with profound clinical significance. We visualise the potential to revolutionise methylation‐based diagnostics upon the effective utilisation of these CpG epi‐marks in conjunction with appropriate PCR‐based methylation techniques. Based on foundational principles and comparative parameters, the liquid biopsy‐based MethyLight assay is established as a highly sensitive, closed‐tube quantitative PCR technique that translates cancer‐specific DNA methylation patterns in clinical samples, marking a transformative development toward minimally invasive cancer diagnostics. Prospective validation of this multiplex assay in a larger, independent cohort of diverse ethnicities and races is essential to confirm its sensitivity, specificity and overall diagnostic accuracy. Furthermore, integrating this method with other minimally invasive liquid biopsy platforms, such as circulating tumour RNA (ctRNA), Circulating Tumour Cells (CTCs) or exosomes, could significantly enhance its clinical utility. Such advancements could pave the way for improved early detection strategies and more personalised treatment plans for ovarian cancer, ultimately transforming patient outcomes and disease management.

## Conflict of interest

No prospective conflicts of interest were disclosed.

## Authors contributions

DB: Research performed the experiments, validation, data curation and analysis, writing—original draft, editing; SG: clinical sample curation, editing and validation; AC: clinical sample curation, editing and troubleshooting; MS*: Conceptualisation, experimentation, supervision, validation, draft: reviewing and editing.

## Supporting information


**Table S1.** Primer and probe sequences for MethyLight Assay.
**Table S2**. CpG wise % distribution of tumour and normal samples for their methylation status.
**Table S3**. Clinicopathological correlation of top hypermethylated CpG sites with their different clinical attributes.
**Table S4**. Distribution of PMR values in tissue and serum cohort.
**Table S5**. Correlation analysis with methylation status of top hypermethylated CpGs and different clinical features using chi square test.
**Fig. S1**. Gel images of different CpG targets along with COBRA and multiplexed gels.
**Fig. S2**. The standard curve plots of top hypermethylated CpGs and endogenous control.
**Fig. S3**. Amplification plots for T1 tumour sample in both tissue and serum cfDNA.

## Data Availability

The data generated in the current study are available from the corresponding author upon request.
