# Supplementary material for: Liquid biopsy‐based diagnostic evaluation of hypermethylated CpG sites for ovarian cancer diagnosis
Source: Mol Oncol. 2026 Jun 16;20(9):2280–95. doi: 10.1002/1878-0261.70277 (PMC13399115; doi:10.1002/1878-0261.70277)
Supplement: Supplementary file 1 [file MOL2-20-2280-s001.docx]

| **Primer/probe** | **5' Modification (Fluorophore)** | **Sequence** | **3' Modification**  **(Quencher)** |
| --- | --- | --- | --- |
| - **cg02957270** | | | |
| **Forward Primer** | None | GGGAAGTTATAATGTTGAAAGGAAATG | None |
| **Reverse_Primer** | None | ACACAATTTAATTTCCTATATCCCTTACTA | None |
| **C_Probe** | /5HEX/ | AC+CR+C+G+AT+TCT | /3IABkFQ/ |
| - **cg10061138** | | | |
| **Forward_Primer** | None | TTGAATGAAATATATTGGGTTCGTAGG | None |
| **Reverse_Primer** | None | CCCCRAAACCTAAACTCTATCA | None |
| **C_Probe** | /5Cy3/ | YGTT+T+A+C+GT+G+TGTT | /3BHQ_2/ |
| - **cg00480298** | | | |
| **Forward_Primer** | None | GGGGTTGATGGTAAAAYGAAGA | None |
| **Reverse_Primer** | None | RTTAACCTAACCCTTCTAACCTAAAA | None |
| **C_Probe** | /5cy5/ | CC+CCR+C+G+AT+A+TA | /3IAbRQSp/ |
| - **Col2A1** | | | |
| **COL2A1 F** | None | TCTAACAATTATAAACTCCAACCACCAA | None |
| **COL2A1 R** | None | GGGAAGATGGGATAGAAGGGAATAT | None |
| **COL2A1-Probe** | /56-FAM/ | CCTTCATTCTAACCCAATACCTATCCCACCTCTAAA | /3IABkFQ/ |

**Table S1 | Primer and probe sequences for MethyLight Assay**. Degenerate Primer and probe sequence with their respective fluorophores and

quenchers for MethyLight test designed and manufactured by IDT technologies.Y=C, T, R=G, A; Grey highlight-degeneracy

**Fig S1 | Gel images of different CpG targets along with COBRA and multiplexed gels.** Representative gels showing targeted CpGs with 50bp ladder A. cg02957270 (116bp) B. cg18878992(121bp) C. cg12910797 (150bp) D. cg10061138(145bp) E. cg10636246 (138bp) F. cg00480298 (178bp) along with their endogenous control (Col2A1) 91bp ; BSTU1 enzyme digestion employing COBRA G. cg02957270 (88+28bp) and H. cg00480298 (150+28bp); Multiplex gels I. Panel 1 cg02957270 (116bp)+cg00480298(178bp)+COL2A1(91bp) J. Panel 2 cg02957270 (116bp)+cg10061138(145bp)+col2a1(91bp)

| **Targeted CpGs** | **Gene** | **Methylated CpG** | | **Unmethylated CpG** | | **Partially methylated CpG** | |
| --- | --- | --- | --- | --- | --- | --- | --- |
|  |  | **Tumour** | **Normal** | **Tumour** | **Normal** | **Tumour** | **Normal** |
| cg18878992 | MAPT | 0 | 0 | 17.78 | 88 | 82.22 | 12 |
| cg00480298 | MAPT | 60 | 20 | 6.67 | 0 | 33.33 | 80 |
| cg02957270 | SKAP1 | 80 | 32 | 0 | 0 | 20 | 68 |
| cg10636246 | AIM2 | 37.78 | 12 | 0 | 0 | 62.22 | 88 |
| cg10061138 | STAB1 | 73.33 | 36 | 0 | 0 | 26.67 | 56 |
| cg12910797 | HOXB3 | 40 | 16 | 0 | 0 | 60 | 84 |

**Table S2 | CpG wise % distribution of tumour and normal samples for their methylation status.** Distribution (%) of tumour and normal tissue

samples by methylation status: methylated, unmethylated, and partially methylated (N = 100; tumour = 65, normal = 35)

|  | **Tissue Characteristics** | | **Sample No.** | | **Target CpG1: cg02957270** | | | **Target CpG2: cg10061138** | | | **Multiplex panel** | | |
| --- | --- | --- | --- | --- | --- | --- | --- | --- | --- | --- | --- | --- | --- |
|  |  |  |  |  |  |  |  |  |  |  | **(cg02957270+cg10061138)** | | |
|  | **Sensitivity and specificity** | | n=100 | | 80%, N/D | | | 73.3%, N/D | | | 82.3%, N/D | | |
|  |  | |  | | **M** | **PM** | | **M** | **PM** | | **Positive** | **Negative** | |
|  | **Tumour** | | 65 | | 52 | 13 | | 47 | 18 | | 53 | 12 | |
|  | **Normal** | | 35 | | 11 | 24 | | 13 | 22 | | 4 | 31 | |
|  | **p-value** | |  | | 0.0000016*** | | | 0.00061766*** | | | 0.00000001*** | | |
|  | **Histology** | |  | | | | | | | | | | |
|  | **Serous** | | 52 | | 43 | 9 | | 36 | 16 | | 46 | 6 | |
|  | **Mucinous** | | 6 | | 3 | 3 | | 4 | 2 | | 4 | 2 | |
|  | **Benign** | | 7 | | 6 | 1 | | 6 | 1 | | 5 | 2 | |
| **p-value** | |  | | ns | | | ns | | | ns | | |  |
|  | **FIGO stage** | |  | |  |  | |  |  | |  |  | |
|  | **I/II** | | 14 | | 14 | 0 | | 8 | 6 | | 10 | 4 | |
|  | **III/IV** | | 51 | | 40 | 11 | | 39 | 12 | | 42 | 9 | |
|  | **p-value** | |  | | ns | | | ns | | | ns | | |
|  | **Age (median = 47)** | |  | |  |  | |  |  | |  |  | |
|  | **≥Median age** | | 52 | | 31 | 21 | | 28 | 24 | | 34 | 18 | |
|  | **<Median age** | | 48 | | 32 | 16 | | 32 | 16 | | 38 | 10 | |
| **p-Value** | |  | | ns | | | ns | | | ns | | |  |
|  | **Menopausal status** | |  | |  |  | |  |  | |  |  | |
|  | **Pre** | | 35 | | 20 | 15 | | 22 | 13 | | 29 | 6 | |
|  | **Post** | | 65 | | 44 | 21 | | 38 | 27 | | 54 | 11 | |
|  | **p-value** | |  | | ns | | | ns | | | ns | | |
|  | **CA125 levels** | |  | |  |  | |  |  | |  |  | |
|  | **<35** | | 20 | | 18 | 2 | | 13 | 7 | | 9 | 11 | |
|  | **≥35** | | 45 | | 33 | 12 | | 35 | 10 | | 31 | 14 | |
|  | **p-value** | |  | | ns | | | ns | | | ns | | |
|  |  | |  | |  | | |  | | |  | | |

**Table S3 | The clinicopathological correlation of top hypermethylated CpG sites with their different clinical attributes.** Clinicopathological correlation of top Hypermethylated targeted CpGs in singleplex and multiplex format of ARMS-PCR and their correlation with the clinicopathological features using chi-square test; ns: Non-significant.

**A. B.**

**C.**

**Fig S2 |** **The standard curve plots of top hypermethylated CpGs and endogenous control.** The standard curves of (A) COL2A1, (B) cg02957270 (C) cg00480298 generated through a multiplex PCR run and tested it on a serial dilution series of methylation-positive normal DNA employing MethyLight test. The standard curves for COL2A1, cg02957270, cg00480298 yielded R² values of 0.9791, 0.9667, and 0.9904, respectively, signifying good reproducibility across standards from different samples.

A. **B.**

**Fig S3 | Amplification plots for T1 tumour sample in both tissue and serum cfDNA.** Amplification plots for the multiplex CpGs 1: Col2A1, 2: cg02957270, 3: cg1006113, and for T1 tumour sample of A. Tissue and B. matched serum sample showing the different Ct Values. The y-axis showing ΔRn normalized fluorescence signal (reporter minus baseline with cycle number on x axis.

| Targeted CpGs |  |  | **Patient no.** | **Min** | **Max** | **Mean ± Std. Error** | **Q1** | **Q2** | **Q3** |
| --- | --- | --- | --- | --- | --- | --- | --- | --- | --- |
| **cg02957270** | Tissue | Tumor | 65 | 2.03 | 93.95 | 21.22 ± 2.39 | 8.47 | 15.6 | 26.31 |
| **cg02957270** |  | Normal | 35 | 0.08 | 7.55 | 2.36 ± 0.32 | 0.44 | 1.35 | 3.56 |
| **cg02957270** | Serum | Tumor | 35 | 1.25 | 68.30 | 11.97± 1.95 | 3.37 | 8.84 | 14.97 |
| **cg02957270** |  | Normal | 20 | 0.12 | 6.12 | 2.04± 0.37 | 0.73 | 1.64 | 2.96 |
| **cg00480298** | Tissue | Tumor | 65 | 0.95 | 65.07 | 20.87 ± 2.23 | 3.71 | 15.95 | 27.33 |
| **cg00480298** |  | Normal | 35 | 0.03 | 9.74 | 3.71 ± 0.47 | 1.42 | 2.88 | 4.46 |
| **cg00480298** | Serum | Tumor | 35 | 0.83 | 23.33 | 7.69 ± 0.86 | 3.73 | 7.38 | 10.66 |
| **cg00480298** |  | Normal | 20 | 0.13 | 7.38 | 2.35 ± 0.49 | 0.67 | 1.55 | 5.62 |
| **cg10061138** | Tissue | Tumor | 65 | 0.25 | 138.51 | 19.26 ± 2.86 | 3.86 | 13.9 | 25.59 |
| **cg10061138** |  | Normal | 35 | 0.19 | 9.91 | 3.47 ± 0.53 | 1.56 | 2.44 | 4.89 |

**Table S4 | Distribution of PMR values in tissue and serum cohort.** PMR values of CpGs (cg02957270 and cg00480297, cg10061138) were quantified in tissue and serum from patients with EOC and a healthy group. PMR stands for percentage of methylated reference. Q1: Quartile 1, Q2: Quartile 2, Q3: Quartile

| **Tissue Characteristics** | **Sample No.** | **Target CpG1: cg02957270** | | **Target CpG2: cg00480298** | | **Multiplex panel** | | **Serum Characteristics** |  | **Target CpG1: cg02957270** | | **Target CpG2: cg00480298** | | **Multiplex panel** | |
| --- | --- | --- | --- | --- | --- | --- | --- | --- | --- | --- | --- | --- | --- | --- | --- |
|  |  |  |  |  |  | **(cg02957270+cg00480298)** | |  |  |  |  |  |  | **(cg02957270+cg00480298)** | |
|  |  | **M** | **U** | **M** | **U** | **Positive** | **Negative** |  |  | **M** | **U** | **M** | **U** | **Positive** | **Negative** |
| **Tumour** | **65** | 55 | 10 | 54 | 11 | 57 | 8 | **Tumour** | **35** | 25 | 10 | 24 | 11 | 32 | 3 |
| **Normal** | **35** | 8 | 27 | 9 | 26 | 6 | 29 | **Normal** | **20** | 3 | 17 | 5 | 15 | 3 | 17 |
| **p-value** |  | < 0.05 | | < 0.05 | | <0.05 | | **p-value** |  | <0.05 | | <0.05 | | <0.05 | |
| **Histology** |  | | | | | | | **Histology** |  | | | | | | |
| **Serous** | **52** | 45 | 7 | 43 | 9 | 46 | 6 | **Serous** | **31** | 22 | 9 | 21 | 10 | 29 | 2 |
| **Mucinous** | **6** | 5 | 1 | 6 | 0 | 6 | 0 | **Mucinous** | **3** | 2 | 1 | 2 | 1 | 2 | 1 |
| **Benign** | **7** | 5 | 2 | 5 | 2 | 5 | 2 | **Benign** | **1** | 1 | 0 | 1 | 0 | 1 | 0 |
| **p-value** |  | ns | | ns | | ns | | **p-value** |  | ns | | ns | | ns | |
| **FIGO stage** |  | | | | | | | **FIGO stage** |  | | | | | | |
| **I/II** | **20** | 16 | 4 | 14 | 6 | 18 | 2 | **I/II** | **15** | 11 | 4 | 11 | 4 | 14 | 0 |
| **III/IV** | **45** | 39 | 6 | 40 | 5 | 39 | 6 | **III/IV** | **20** | 14 | 6 | 13 | 7 | 18 | 2 |
| **p-value** |  | ns | | ns | | ns | | **p-value** |  | ns | | ns | | ns | |
| **Age (median = 47)** |  | | | | | | | **Age (median = 47)** |  | | | | | | |
| **≥Median age** | **52** | 35 | 17 | 32 | 20 | 31 | 21 | **≥Median age** | **29** | 12 | 17 | 18 | 11 | 21 | 8 |
| **<Median age** | **48** | 28 | 20 | 31 | 17 | 32 | 16 | **<Median age** | **26** | 16 | 10 | 11 | 15 | 14 | 12 |
| **p-Value** |  | ns | | ns | | ns | | **p-Value** |  | ns | | ns | | ns | |
| **Menopausal status** |  | | | | | | | **Menopausal status** |  | | | | | | |
| **Pre** | **35** | 28 | 7 | 29 | 6 | 28 | 7 | **Pre** | **22** | 15 | 7 | 17 | 5 | 21 | 1 |
| **Post** | **65** | 36 | 29 | 39 | 26 | 34 | 31 | **Post** | **33** | 13 | 20 | 12 | 21 | 14 | 19 |
| **p-value** |  | < 0.05 | | < 0.05 | | < 0.05 | | **p-value** |  | <0.05 | | <0.05 | | <0.05 | |
| **CA125 levels** |  | | | | | | | **CA125 levels** |  | | | | | | |
| **<35** | **20** | 17 | 4 | 14 | 6 | 19 | 1 | **<35** | **10** | 8 | 2 | 6 | 4 | 9 | 1 |
| **≥35** | **45** | 38 | 6 | 40 | 5 | 38 | 7 | **≥35** | **25** | 17 | 8 | 18 | 7 | 23 | 2 |
| **p-value** |  | ns | | ns | | ns | | **p-value** |  | ns | | ns | | ns | |

**Table S5 | Correlation analysis with methylation status of top hypermethylated CpGs and different clinical features using chi square test:** Clinicopathological correlation of top two Hypermethylated targeted CpGs of MethyLight test in singleplex and multiplex format and their correlation with the clinicopathological correlation using chi-square test
